# Impaired Resting-State Functional Integrations within Default Mode Network of Generalized Tonic-Clonic Seizures Epilepsy

**DOI:** 10.1371/journal.pone.0017294

**Published:** 2011-02-25

**Authors:** Ming Song, Hanjian Du, Nan Wu, Bing Hou, Guocai Wu, Jian Wang, Hua Feng, Tianzi Jiang

**Affiliations:** 1 National Laboratory of Pattern Recognition, Institute of Automation, Chinese Academy of Sciences, Beijing, China; 2 Department of Neurosurgery, Southwest Hospital, Third Military Medical University, Chongqing, China; 3 Department of Radiology, Southwest Hospital, Third Military Medical University, Chongqing, China; 4 Key Laboratory for NeuroInformation of Ministry of Education, School of Life Science and Technology, University of Electronic Science and Technology of China, Chengdu, China; Newcastle University, United Kingdom

## Abstract

Generalized tonic-clonic seizures (GTCS) are characterized by unresponsiveness and convulsions, which cause complete loss of consciousness. Many recent studies have found that the ictal alterations in brain activity of the GTCS epilepsy patients are focally involved in some brain regions, including thalamus, upper brainstem, medial prefrontal cortex, posterior midbrain regions, and lateral parietal cortex. Notably, many of these affected brain regions are the same and overlap considerably with the components of the so-called default mode network (DMN). Here, we hypothesize that the brain activity of the DMN of the GTCS epilepsy patients are different from normal controls, even in the resting state. To test this hypothesis, we compared the DMN of the GTCS epilepsy patients and the controls using the resting state functional magnetic resonance imaging. Thirteen brain areas in the DMN were extracted, and a complete undirected weighted graph was used to model the DMN for each participant. When directly comparing the edges of the graph, we found significant decreased functional connectivities within the DMN of the GTCS epilepsy patients comparing to the controls. As for the nodes of the graph, we found that the degree of some brain areas within the DMN was significantly reduced in the GTCS epilepsy patients, including the anterior medial prefrontal cortex, the bilateral superior frontal cortex, and the posterior cingulate cortex. Then we investigated into possible mechanisms of how GTCS epilepsy could cause the reduction of the functional integrations of DMN. We suggested the damaged functional integrations of the DMN in the GTCS epilepsy patients even during the resting state, which could help to understand the neural correlations of the impaired consciousness of GTCS epilepsy patients.

## Introduction

A consistent network of human brain regions, including medial prefrontal cortex, posterior midbrain regions, medial temporal lobes and lateral parietal cortex, showed high levels of activity when no explicit task was performed [1], [2]. It has been suggested that the human brain has a default or intrinsic mode of functioning and these brain regions constitute so-called default mode network (DMN) [3], [4]. Although there were some debates about cognitive functions of the DMN [5], [6], [7], some investigators suggest that the DMN directly contributes to internal mentation that is largely detached from the external world, including self-reflective thoughts and judgments, conceiving the mental states of other peoples and envisioning the future to make up alternative decision [8]. In particular, recent studies have found the activity of the DMN are closely associated with some specific consciousness states, such as anesthesia [9] and sleep [10], [11], [12]. For example, in humans during deep sleep, which is characterized by disengagement of the consciousness, the interactions between the components of the DMN are found to be reduced, especially in the medial prefrontal cortex [11].

Though notoriously difficult to define, consciousness is central to many neurological conditions, ranging from neurodegenerative dementias to coma and vegetative states, and from sleep disorders to epilepsy [13]. In fact, the damaged activity of the DMN has been found in some neuropsychiatric diseases (for review, see [14]). Generalized tonic-clonic seizures (GTCS) are characterized by unresponsiveness and convulsions, which cause complete loss of consciousness. Many recent studies have found that the ictal alterations in brain activity of the GTCS epileptic patients are focally involved in some brain regions, including thalamus, upper brainstem, medial prefrontal cortex, posterior midbrain regions and lateral parietal cortex [15], [16]. Notably, many of these affected brain regions are the same and overlap considerably with the components of the DMN. Based on evidences from neurophysiology, brain imaging and pathology together, some researchers believed that the abnormal brain activity in the DMN might be the neural correlates of the complete impaired consciousness of the GTCS epilepsy patients during the seizures [13].

Functional connectivity (FC), which studies temporal correlations between the signals in different brain regions, has been widely used in functional magnetic resonance imaging (fMRI) studies to investigate direct or indirect interactions between brain regions [17]. Recent progress in complex brain networks, based largely on graph theory, provides a powerful method to study the brain network organization [18]. When describing a specific brain network, a graph can provide an abstract representation for the brain areas within the network and their interaction.

In the present study, we hypothesize that the brain activity of the DMN of the GTCS epilepsy patients may be different from one of the normal controls, even in the resting state. To test this hypothesis, using the graph theory, we compared the DMN of the GTCS epilepsy patients and the controls with the resting state fMRI data.

## Materials and Methods

### Study population

The study involved 14 GTCS epilepsy patients and 29 healthy normal subjects. All participants were male and right-handed. Fourteen epilepsy patients were recruited from the department of neurosurgery of Southwest Hospital of the Third Military Medical University, and 29 controls were recruited by advertisement. There was no significant difference in age between the two groups (two sample t-test, P>0.05; GTCS epilepsy patients: age = 26.1±6.1 years; control: age = 27.1±4.5 years). All participants gave written informed consent. This study was approved by the ethical committee of the Third Military Medical University.

The clinical information of the GTCS epilepsy patients, including The National Hospital Seizure Severity Scale (NHS3), was collected through interviews with the patients and their relatives who had witnessed the patient's epileptic seizures. Any abnormity had not been detected for all of the patients in routine MRI examinations. None of the patients reported a history of drug intoxication, encephalopathy, or brain trauma. However, an experienced EEG physician found that all the patients showed generalized spike and wave or polyspikes discharges against a normal background in Video-EEG monitoring. Based on their seizure history, their seizure semiology and results from video-EEG recording, these patients were diagnosed as idiopathic generalized epilepsy (IGE) with only generalized tonic-clonic seizures according to the International League Against Epilepsy (ILAE) classification. More information about the patients was shown in Table 1.

**Table 1:** Clinical information of GTCS epilepsy patients.

Patient	Age (years)	Onset (years)	Duration (years)	Antiepileptic drugs	NHS3
1	34	22	12	VPA	10
2	36	16	20	PHT	13
3	35	19	16	VPA	12
4	28	15	13	VPA	15
5	27	17	10	CBZ/PHT	12
6	19	15	4	VPA/CBZ	10
7	25	23	2	VPA	13
8	32	22	10	CBZ	15
9	21	17	4	CBZ	17
10	20	6	14	PHT	12
11	19	10	9	CBZ	14
12	24	20	4	PB	10
13	20	15	5	CBZ/PHT	14
14	25	9	16	CBZ/VPA	10

Abbreviations: sodium valproate (VPA), carbamazepine (CBZ), phenytoin (PHT), Phenobarbital (PB), The National Hospital Seizure Severity Scale (NHS3).

Twenty-nine controls underwent a comprehensive MRI brain examination to ensure that they had no neurological lesions. All controls had no history of neurological or systemic illness, head injury, and drug or alcohol abuse.

### Scan acquisition

MR imaging was carried out using a 3.0-Tesla MR scanner (Magnetom Trio, Siemens, Erlangen, Germany). Functional images were collected axially by using an echo-planar imaging (EPI) sequence sensitive to BOLD contrast. The acquisition parameters were as follows: 36 slices, 2000/30 ms (TR/TE), 3.0/1.0 mm (thickness/gap), 192
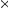
192 mm (FOV), 64
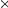
64 (resolution within slice), 90° (flip angle). The FOV covered all of brain regions for all participants. During the resting state scanning, the participants were instructed to keep still with their eyes closed, as motionless as possible and not to think about anything in particular. For each participant, the fMRI scan during the resting state lasted for 8 min and 240 volumes were obtained.

Additionally, anatomical image datasets were also acquired with a standard T1-weighted high-resolution anatomic scan of a magnetization-prepared rapid gradient echo (MPRAGE) sequence for each participant in this study.

### Data preprocessing

Image preprocessing was conducted using SPM2 (http://www.fil.ion.ucl.ac.uk/spm). The first 10 volumes of each participant were discarded. The remaining 230 functional scans were first corrected for within-scan acquisition time differences between slices and realigned to the first volume to correct for inter-scan movements. Next, the functional scans were spatially normalized to a standard EPI template and resampled to the voxel size of 3
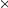
3
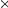
3 mm. Subsequently, the functional images were spatially smoothed with a Gaussian kernel of 4
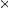
4
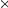
4 mm full-width at half maximum. Then, linear regression was used to remove the influence of head motion, whole brain signals and linear trends [19], [20], [21], [22]. Finally, to reduce low-frequency drift and high-frequency noise, the fMRI data were temporally band-pass filtered (0.01–0.08 Hz) with AFNI (http://afni.nimh.nih.gov/). The parameters obtained during movement correction showed that the maximum displacement in the cardinal direction was not greater than 1 mm, and the maximum spin was not greater than 1° for each participant.

### Region definition

Although the core regions associated with the brain's DMN have been identified, the precise components of the DMN and their loci are still not very clear [8]. In the present study, we used a priori regions of interest (ROIs) to define the DMN as previous studies [23], [24], [25], [26]. For details about the ROI definition, please refer to [27]. The coordinates of a priori ROIs in the present study were shown in Table 2. All ROIs were defined as a spherical region with a radius of 6mm at the center of the obtained coordinates of a priori ROI. Since the size of voxel in the present study was 3
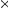
3
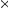
3 mm, each ROI was comprised of 33 voxels.

**Table 2:** Seed regions for the DMN.

Brain region	Abbreviations	MNI Coordinates	BA
Medial prefrontal cortex (anterior)	aMPFC	(−3,57,21)	10
Left superior frontal cortex	L.Sup.F	(−12, 45,48)	8/9
Right superior frontal cortex	R.Sup.F	(21,42,48)	8/9
Ventral anterior cingulate cortex	vACC	(−3,36,−6)	32
Left inferior temporal cortex	L.IT	(−54,−3,−30)	20/21
Right inferior temporal cortex	R.IT	(54,0,−30)	20/21
Left parahippocampal gyrus	L.PHC	(−24,−18,−27)	35/36
Right parahippocampal gyrus	R.PHC	(24,−12,−27)	35/36
Posterior cingulate cortex	PCC	(−3,−45,33)	31
Retrosplenial	Rsp	(−15,−54,6)	29/30
Left lateral parietal cortex	L.LatP	(−54,−69,36)	39/40
Right lateral parietal cortex	R.LatP	(54,−63,33)	39/40
Cerebellar tonsils	Cereb	(9,−51,−45)	-

### Subject-level DMN graph generation

After extracting the 13 ROIs for each participant, we computed the functional connectivity between each pair of the 13 ROIs. For each pair of ROIs, the functional connectivity between them was generated by averaging the BOLD time series separately in the two regions, and then computing the Pearson's correlation coefficient between the two averaged time series. The resulting correlation was then transformed to approximate Gaussian distribution using Fisher's r-to-z transformation. Thus, for each participant, we obtained a 13
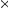
13 matrix, with each element representing the strength of functional connectivity between the corresponding two brain regions within the DMN.

Here, we used the undirected weighted graph to model the DMN. Specifically, the node of graph was used to denote the brain region within the DMN, and the weight of the edge between two nodes represented the z-valued strength of functional connectivity between the corresponding two brain regions. Thus, we constructed a complete undirected weighted graph to model the topology of the DMN for each participant.

### Degree of node in graph

In graph theory, the degree s_i_ of a node i was the number of edges linking to the node, and was defined as:
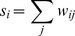
(1)where w_ij_ denoted the weighted edge that connected node i and node j, that is, in the present study, the z-valued strength of the functional connectivity between brain region i and brain region j. The degree s_i_ can be used to qualify the extent to which the node was central in the graph. With the node degree, we can define the hub node, which is the node with high degree in a graph.

In the present study, by comparing the edges of the graph, we can investigate the difference of functional connectivity between any pair of brain areas. On the other hand, by comparing the degree of node in the graph, we can investigate the centrality of individual brain area within DMN. From equation 1, it is easy to see that the connection strength and the node degree are not independent. However, we think that investigating the connection strength and the node degree can, to some extent, provide some different aspects of information about the functional integrations of brain network.

### Group-level DMN graph analysis

First, using one-sample t test, we checked whether all functional connectivities within the DMN were significantly greater than 0 in each of the two groups. Then, we used two-sample t test to investigate whether there was significant difference in the functional connectivity within the DMN and the degree of each brain area of DMN between the two groups. Here, we used the Benjamini and Hochberg False Discovery Rate to correct the multiple comparisons [28]. Finally, we investigated whether there was the significant Pearson's correlation between the strength of the functional connectivity and the NHS3 score in the GTCS epilepsy patients group.

## Results

Using the T1-weighted MRI data, we have not found any significant difference in voxel-based morphology and cortical thickness between the GTCS patients and the controls.

**Figure 1:**
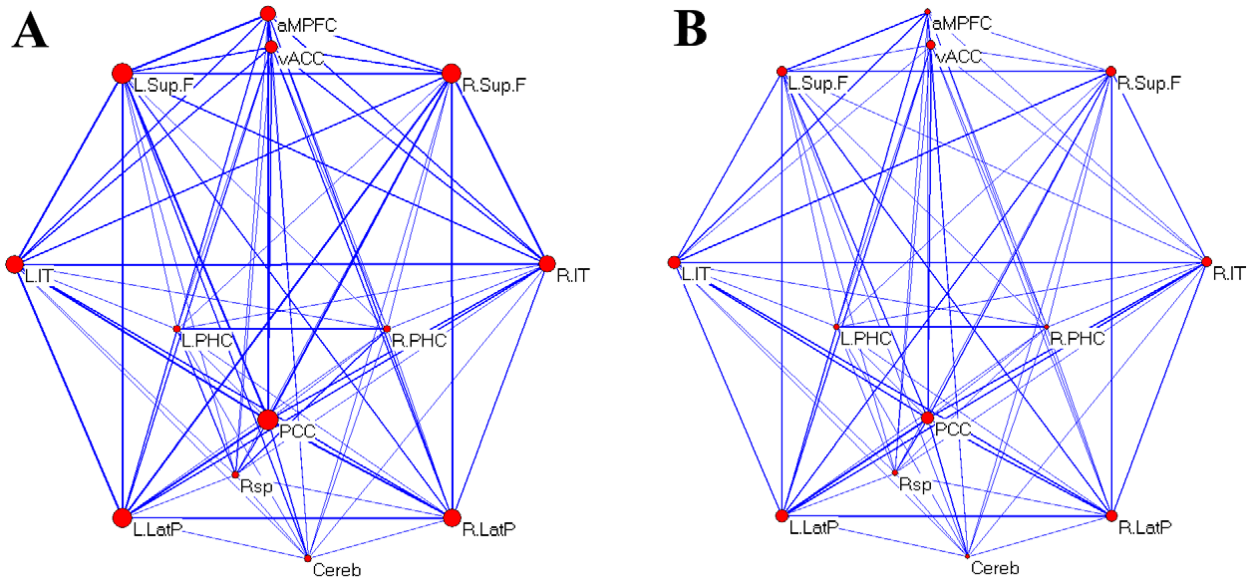
The mean functional connectivity graph of the DMN in a pseudoanatomical organization separately for the controls group (A) and the GTCS epilepsy patients group (B). Line width is proportional to the mean connection strength between any pair of brain regions within the DMN, and node size is proportional to the mean degree of the node.

In functional connectivity analysis, we found that all functional connectivities within the DMN were significantly greater than 0 in the control group (P<0.05, corrected). The mean DMN graphs respectively for the two groups were shown in Figure 1. As shown in Figure 1, the homologous bilateral brain regions showed strong functional connectivity, and the PCC showed the high degree.

**Figure 2:**
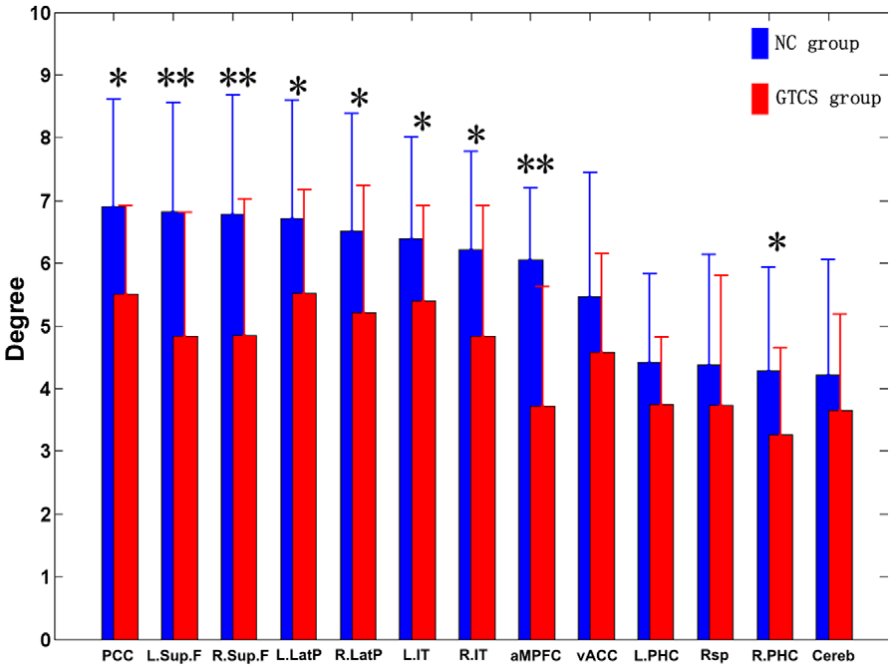
The comparison of the degree for each brain region within the DMN of the two groups. The “*” denoted the significantly different between the GTCS epilepsy patients group and the controls group (p<0.05 corrected). The “**” denoted the significantly different between the GTCS epilepsy patients group and the normal control group (p<0.01 corrected). The brain regions in the x axis were sorted by the average degree in the normal control group.

Comparing the strength of the functional connectivity within the DMN between the two groups, we found significantly decreased functional connectivity in the GTCS epilepsy patients group (P<0.05, corrected), as shown in Table 3. We did not find any increased functional connectivity for the GTCS epilepsy patients in comparison to the controls. Notably, most of the significantly decreased functional connectivities were restricted to the brain regions in the prefrontal cortex, such as the anterior medial prefrontal cortex (aMPFC) and the bilateral superior frontal cortex. Additionally, the degree of many brain areas within the DMN was significantly reduced (P<0.05, corrected), including the aMPFC, bilateral superior frontal cortex, posterior cingulate cortex, bilateral parietal cortex and bilateral inferior temporal cortex. These results about the node degree were shown in Figure 2.

**Table 3:** The significantly different functional connectivity within the DMN between the controls group and the GTCS epilepsy patients group.

Functional connectivity		NC group	GTCS group(mean±std)	P value*
Brain area 1	Brain area 2	(mean±std)		
aMPFC	L.Sup.Frontal	0.6453±0.2341	0.3932±0.1916	0.0006
aMPFC	R.Sup.Frontal	0.5161±0.2426	0.2058±0.2055	0.0001
aMPFC	vACC	0.5722±0.2500	0.3111±0.2295	0.001
aMPFC	R.IT	0.4873±0.2029	0.2034±0.2417	0.0001
aMPFC	PCC	0.5781±0.2124	0.2945±0.2639	0.0002
aMPFC	L.LatP	0.5448±0.1649	0.3375±0.2887	0.0023
aMPFC	R.LatP	0.3879±0.1913	0.1493±0.2416	0.0005
L.Sup.Frontal	R.IT	0.4914±0.2170	0.2336±0.2661	0.0008
L.Sup.Frontal	PCC	0.6254±0.2258	0.4125±0.2274	0.0031
R.Sup.Frontal	R.IT	0.5622±0.2572	0.3436±0.2567	0.0062
R.Sup.Frontal	L.LatP	0.6340±0.2868	0.3655±0.2937	0.0034
PCC	L.IT	0.6249±0.2086	0.4549±0.1462	0.0046

The functional connectivity was expressed as z-scored Fisher-coefficients for testing pair-wise correlations of BOLD signal between the corresponding two nodes.

*, P value was corrected for multiple comparisons.

Additionally, we found that the strength of the functional connectivity between the aMPFC and the ventral anterior cingulate cortex (vACC) was significantly correlated to the NHS3 score of the GTCS epilepsy patients (Pearson's r = −0.64, P<0.05, uncorrected).

## Discussion

As mentioned in the introduction, many studies have found that the brain activities of some brain areas in DMN are abnormal for the GTCS epilepsy patients, whereas most of these studies focused on the ictal alterations in brain activity. The present study used the resting state fMRI and the graph theory to investigate the functional integrations of DMN for GTCS epilepsy patients. We found significantly decreased functional connectivity within the DMN of the GTCS epilepsy patients in comparison to the controls. Additionally, the degree of some brain areas within the DMN was significantly reduced. Taken together, these results suggest the reduced functional integrations of the DMN in the GTCS epilepsy patients, even in resting state.

Single-photon emission computed tomography (SPECT) studies found that cerebral blood flow (CBF) decreases occurred during and following secondarily generalized tonic-clonic seizures in the medial prefrontal cortex, posterior cingulate gyrus and lateral parietal cortex, overlapping the core elements of the DMN. Concurrently, analysis of patient behavior showed impaired consciousness during and following seizures [15]. The studies of EEG-fMRI simultaneous recording found, for the generalized epilepsy patients (including GTCS and absence seizures), when the generalized spike wave was seen in the EEG, the posterior cingulated gyrus, precuneus, and the lateral sides of the parietal cortex showed a negative activation, and the medial frontal brain cortex showed abnormal activities [16], [29], [30], [31]. Additionally, in a growing resting state fMRI studies the disruption of functional connectivity networks in epilepsy have been reported [32], [33]. Recently, Zhang et al. used data-driven independent component analysis to investigate the alterations of the DMN in the mesial temporal lobe epilepsy with complex partial seizure. They found that the activities of DMN, especially in medial prefrontal cortex, significantly decreased in the epilepsy patients [34]. These evidences from various modalities of neuroimaging, together with the present study, suggest disrupted network organization of the DMN in consciousness impaired epilepsy patients.

Here, we think that there are the following possibilities to cause the reduction of functional integrations of the DMN in the GTCS epilepsy patients.

### Chronic epilepsy impair intrinsic brain activity of the DMN of GTCS epilepsy patients

More and more evidence show that the “resting state” may be much more than a “noise background”, and it is likely to reflect a default or intrinsic functioning mode of human brain [7]. The generalized tonic-clonic seizures are characterized by a complete impaired consciousness [13]. Recently, Vanhaudenhuyse investigated whether the integrity of the resting state connectivity pattern in the DMN would differ in different pathological alterations of consciousness, from locked-in syndrome to minimally conscious, vegetative then coma patients. They found that the functional connectivity pattern in the DMN was decreased in severely brain-damaged patients, in proportion to their degree of consciousness impairment [35]. Although the pathological foci of idiopathic epilepsy are not yet clear, epileptic discharges can be propagated through nerve fibers. We speculated that chronic epileptic discharges which can cause consciousness impairment might damage intrinsic brain activity of the DMN in GTCS epilepsy patients.

### Interictal epileptic discharges during scanning trigger the transient changes of DMN

It is possible that the interictal discharges, which appears frequently during the resting state fMRI scanning, would reduce functional integrations of the DMN. The recent EEG-fMRI simultaneous recording studies found, when the generalized spike wave appeared, the posterior cingulated gyrus, precuneus, and the lateral parietal cortex showed a negative activation, whereas the medial frontal cortex showed incomplete synchronized activities [16]. The incomplete synchronized activities could decrease the functional connectivity between the brain areas, especially between the medial prefrontal cortex and the posterior midbrain regions. So, interictal epilepsy activity during scanning might one of the reasons for the reduced functional integrations of the DMN in the present study.

### Antiepileptic medications suppress ongoing activity of DMN

The epilepsy patients in the present study take some antiepileptic medications, most of which are the first generation of antiepileptic drugs and derived from sedatives. These drugs can cause some side effect on physiological processes, including neurotransmission and metabolism, and further damage the cognitive ability [36], [37]. So, it is possible that antiepileptic medications suppress ongoing activity of the DMN, and thus reduce functional integrations of the DMN. However, in the present study, we found that the degree of a few brain areas within the DMN was reduced, instead of all brain areas within the DMN. Therefore, we think that antiepileptic drugs are not likely to be the sole explanation for the present results.

### Functional reorganization and plasticity of DMN

Chronic epilepsy generally impairs cognition, but it can also cause functional reorganization and plasticity [37]. Notably, the onset of epileptic seizure was during the period of children or adolescence for 9 of 14 patients in the present study. Some studies suggested that the DMN's functioning could be achieved by charactering its development, that is, the functional integrations of the DMN could be comparatively weak at children age, and then gradually stronger over development [38]. So, it is possible that early epileptic seizures of the GTCS epilepsy patients disturb the development of the DMN, and change the structure of the DMN. In the present study, we used a priori ROIs to define the DMN. The coordinates of a priori ROIs were obtained from previous studies and our own experience. The error of the coordinates of a priori ROIs, regardless from the analysis computation or the functional reorganization, is likely to reduce the functional connectivity within the DMN. However, Zhang et al. used data-driven independent component analysis (ICA) to investigate the alterations of the DMN in the mesial temporal lobe epilepsy with complex partial seizure [34]. The ICA does not require defining the ROI. And, Zhang et al. found the consistent results with the present study, which indicated that the error of the coordinates of a priori ROIs is not likely to change our findings.

Summarily, we prefer to think that the four mechanisms might act together to cause the reduction of the functional integrations of the DMN in the GTCS epilepsy patients. In future, we plan to use EEG-fMRI simultaneous recording technology to classify the resting state fMRI scanning according to whether the epileptiform waves exist or not. Thus, we can identify whether the reduced functional integrations of the DMN in the GTCS epilepsy patients would result from the impaired intrinsic brain activity or the incomplete synchronized brain activities during the epilepsy activity. Additionally, there is no wide agreement of the composition of the DMN, and we used a priori regions of interest to define the DMN in the present study. A complete undirected weighted graph with 13 nodes gives an approximate picture to the DMN. As the study of the DMN goes in depth, we believe that there will be more appropriate methods for modeling the DMN.

### Conclusions

In this study, we found significantly decreased functional connectivity within the DMN of the GTCS epilepsy patients in comparison to the controls. Additionally the degree of some brain areas within the DMN was significantly reduced. It is possible that several mechanisms might explain these findings. However, these results suggest the reduced functional integrations of the DMN in the GTCS epilepsy patients, which could be helpful to understand the neural correlation of the impaired consciousness of GTCS epilepsy patients.
